# Rapid and Visual Differentiation of *Mycobacterium tuberculosis* From the *Mycobacterium tuberculosis* Complex Using Multiplex Loop-Mediated Isothermal Amplification Coupled With a Nanoparticle-Based Lateral Flow Biosensor

**DOI:** 10.3389/fmicb.2021.708658

**Published:** 2021-08-02

**Authors:** Xinggui Yang, Junfei Huang, Xu Chen, Ziyu Xiao, Xiaojuan Wang, Yijiang Chen, Wenlin Zheng, Wei Chen, Huijuan Chen, Shiguang Lei, Yong Hu, Shijun Li

**Affiliations:** ^1^Public Health School, Guizhou Medical University, Guiyang, China; ^2^Guizhou Provincial Center for Disease Control and Prevention, Guiyang, China; ^3^The Second Affiliated Hospital, Guizhou University of Traditional Chinese Medicine, Guiyang, China

**Keywords:** *Mycobacterium tuberculosis*, loop-mediated isothermal amplification, mLAMP, lateral flow biosensor, MTB

## Abstract

Tuberculosis (TB) is a chronic infectious disease mainly caused by *Mycobacterium tuberculosis* (MTB), but other members of the *Mycobacterium tuberculosis* complex (MTBC), especially *Mycobacterium bovis* (pyrazinamide-resistant organisms), may also be involved. Thus, the ability to rapidly detect and identify MTB from other MTBC members (e.g., *M. bovis*, *Mycobacterium microti*, *Mycobacterium africanum*) is essential for the prevention and treatment of TB. A novel diagnostic method for the rapid detection and differentiation of MTB, which employs multiplex loop-mediated isothermal amplification (mLAMP) combined with a nanoparticle-based lateral flow biosensor (LFB), was established (mLAMP-LFB). Two sets of specific primers that target the *IS6110* and *mtp40* genes were designed according to the principle of LAMP. Various pathogens were used to optimize and evaluate the mLAMP-LFB assay. The optimal conditions for mLAMP-LFB were determined to be 66°C and 40 min, and the amplicons were directly verified by observing the test lines on the biosensor. The LAMP assay limit of detection (LoD) was 125 fg per vessel for the pure genomic DNA of MTB and 4.8 × 10^3^ CFU/ml for the sputum samples, and the analytical specificity was 100%. In addition, the whole process, including the clinical specimen processing (35 min), isothermal amplification (40 min), and result confirmation (1–2 min), could be completed in approximately 80 min. Thus, mLAMP-LFB is a rapid, reliable, and sensitive method that is able to detect representative members of MTBC and simultaneously differentiate MTB from other MTBC members, and it can be used as a potential screening tool for TB in clinical, field, and basic laboratory settings.

## Introduction

Tuberculosis (TB) is a chronic infectious disease mainly caused by *Mycobacterium tuberculosis* (MTB), but other members of the *Mycobacterium tuberculosis* complex (MTBC), especially *Mycobacterium bovis*, may also be involved (Ansumana et al., 2017; Scott et al., 2017). In 2016, approximately 10.4 million new TB cases (most of the cases were MTB infections) were reported globally, of which 147,000 new zoonotic TB cases (mainly *M. bovis*) accounted for 1.4% of the global incidences of TB (WHO, 2016; Devi et al., 2021). However, the true prevalence of zoonotic TB in the world is probably underestimated because it is difficult to accurately distinguish MTB and *M. bovis* in actual diagnoses (WHO, 2016; Devi et al., 2021). In particular, the treatment period and antituberculosis drugs for TB caused by MTB infections are different from those used for *M. bovis* (pyrazinamide-resistant organisms) infection (Scott et al., 2017). Thus, the ability to rapidly detect and differentiate MTB from other MTBC members (e.g., *M. bovis*, *Mycobacterium microti*, *Mycobacterium africanum*) is essential for the prevention and treatment of TB (Spositto et al., 2014).

Currently, conventional methods, including acid-fast bacilli (AFB) smears, solid cultures (Lowenstein–Jensen medium, L-J), biochemical tests, and immunological tests (e.g., T-SOPT TB and enzyme-linked immunosorbent assays), are mainly used in clinical examinations to detect MTBC strains (Otu et al., 2008; Rodriguez-Morales and Castañeda-Hernández, 2014; Agrawal et al., 2016; Lyashchenko et al., 2020). In particular, the T-SPOT TB assay, which is based on the detection of MTB-specific interferon-γ-secreting T cells in peripheral blood mononuclear cells, is widely used to detect MTB infections (Rao et al., 2021). Nevertheless, it is difficult to meet the sensitivity and time requirements necessary for rapid examination using the abovementioned methods (Otu et al., 2008; Wadhwa et al., 2014; Agrawal et al., 2016; Lyashchenko et al., 2020). Thus, quick and sensitive diagnostic techniques are required for the detection and differentiation of specific MTBC members in populations.

In recent years, various molecular techniques have been widely applied to rapidly recognize MTBC strains (Nghiem et al., 2015; Agrawal et al., 2016). Polymerase chain reaction (PCR) and PCR-based assays (e.g., multiplex PCR, real-time quantitative PCR, and GeneXpert methods) have also been reported as the most commonly performed methods in past studies (Nghiem et al., 2015; Gallo et al., 2016; Lekhak et al., 2016). Among them, GeneXpert, which utilizes the DNA-PCR technique to simultaneously detect MTB and rifampicin resistance-related mutations, is widely used in the diagnosis of TB (Agrawal et al., 2016). Although these methods have outstanding analytical capabilities, the requirements of special apparatuses (thermal cycling instruments), poor availability (reactions require expensive reagents), and long detection procedures (2–4 h) restrict their application in point-of-care and field laboratories (Gallo et al., 2016; Lekhak et al., 2016).

To overcome these disadvantages posed by PCR-based techniques, isothermal amplification-based diagnostic tests have been developed for the diagnosis of TB (Song et al., 2021). Loop-mediated isothermal amplification (LAMP), which requires multiple primers (four core primers and/or two loop primers) recognizing six and/or eight regions on target sequences, was developed to achieve high-efficiency amplification of nucleic acids (Wang et al., 2017; Wong et al., 2018). Detection indicators (e.g., malachite green, hydroxy naphthol blue, and SYBR Green) have been employed for monitoring the LAMP reaction, and the LAMP tests can be judged directly by the naked eye when the LAMP reactions are incubated for approximately 40–60 min at a fixed temperature (N’guessan et al., 2016; Wang et al., 2017). In particular, detection of these visual indicators is easy to perform and convenient in the LAMP procedure. The whole test process of the LAMP test does not rely on special instruments, and thus, it has been applied in the detection of various pathogens, including bacteria, viruses, fungi, and emerging/re-emerging infectious agents (Chen et al., 2015; Huang et al., 2020; Schermer et al., 2020).

So far, the studies that have developed LAMP-based diagnosis for TB have usually detected a specific molecular marker (e.g., *IS6110*, *mpb64*, *IS1081*, and *gyrB*); thus, it is difficult to accurately differentiate TB that is infected by MTB or other MTBC members (especially *M. bovis*) (Aryan et al., 2010; Sharma et al., 2016, 2017; Joon et al., 2017; Wang et al., 2021). Within the mycobacterial genes, several target sequences have been confirmed to be able to detect all representative MTBC strains (targeting the *IS6110* sequence with a widely used and conventional method is more efficient for diagnosing TB due to its multiple copies in the MTBC genome) (Joon et al., 2017). Meanwhile, a commonly applied and differentiating approach targeting the *mtp40* element in accurate diagnosis tests and methods based on the *mtp40* gene (e.g., PCR and nested PCR assay) can identify TB caused by MTB (Jia et al., 2012; Kathirvel et al., 2014; Chu et al., 2019). However, this report focuses on how to sufficiently utilize the *IS6110* and *mtp40* genes in combination with the LAMP assay (one-step method) to achieve rapid, accurate, sensitive differentiation of MTB from other MTBC members to simplify the detection process. Thus, a visual, sensitive, and target-specific nanoparticle-based lateral flow biosensor (LFB) was successfully designed and applied, which can be utilized for the verification of the two amplification products of nucleic acid labeling (Wang et al., 2017; Chen et al., 2019). The method effectively overcomes the disadvantages of other confirmation methods for mLAMP amplicons (e.g., agarose gel electrophoresis, real-time turbidimetry, and visual indicators) and shortens the time of the verification procedure (1–2 min) (Li et al., 2019).

In this report, we employed a multiplex LAMP assay combined with an LFB strip (mLAMP-LFB) to detect representative bacteria, including MTBC members, nontuberculous mycobacterium (NTM), and nonmycobacteria, and to simultaneously differentiate MTB from other MTBC members. In the mLAMP-LFB system, target genes (*IS6110* and *mtp40*) were amplified in a LAMP reaction tube, and the results were visualized using LFB. Pure culture and clinical sputum samples were used to optimize the mLAMP-LFB assay and evaluate the specificity, sensitivity, and feasibility of the TB-mLAMP-LFB method.

## Materials and Methods

### Ethics Statement

The study was approved by the Human Ethics Committee of the Guizhou Provincial Center for Disease Control and Prevention and complied with the Declaration of Helsinki. All data/isolates were analyzed anonymously.

### Reagents and Apparatus

DNA isothermal amplification kits and visual detection reagent (Malachite Green, MG) were purchased from Bei-Jing HaiTaiZhengYuan Co., Ltd. (Beijing, China). The Bacterial Genomic DNA Extraction Kit was provided by Takara Biomedical Technology Co., Ltd. (Beijing, China). Biotin-14-dCTP was provided by Tian-Jin Huidexin Technology Development Co., Ltd. (Tianjin, China). The backing card, sample pad, conjugate pad, nitrocellulose membrane (NC), and absorbent pad were purchased from Jie-Yi Biotechnology Co., Ltd. (Shanghai, China). Biotinylated bovine serum albumin (biotin-BSA), rabbit anti-fluorescein antibody (anti-FITC), and sheep anti-digoxigenin antibody (anti-Dig) were purchased from Abcam Co., Ltd. (Shanghai, China). Dye (crimson red) streptavidin-coated polymer nanoparticles (129 nm, 10 mg/ml, 100 mM borate, pH 8.5 with 0.1% BSA, 0.05% Tween 20, and 10 mM EDTA) were obtained from Bangs Laboratories, Inc. (Fishers, IN, United States).

### Preparation of the Nanoparticle-Based Lateral Flow Biosensor

The biosensor (60 mm × 4 mm) was constructed with diminutive modifications based on previous reports (Wang et al., 2017; Li et al., 2020). Briefly, the sample pad, conjugate pad, NC membrane, and absorbent pad were laminated onto a plastic adhesive backing card. Then, the biotin-BSA (2.5 mg/ml), anti-FITC (0.15 mg/ml), and anti-Dig (0.2 mg/ml) conjugates were immobilized on the NC membrane. Thus, there were three bands containing the CL (control line, conjugated with biotin-BSA), TL1 (test line 1, conjugated with anti-FITC), and TL2 (test line 2, conjugated with anti-Dig), which were separated by 5 mm. Dye streptavidin-coated polymer nanoparticles (SA-PNPs) were collected in the conjugate pad. For this study, following our design, the LFB was manufactured by Tian-Jin HuiDeXin Biotech Co., Ltd. (Tianjin, China). The LFB was stored in a dry and dark place at 4°C. The detection method of LFB involves depositing an aliquot (1.2 μl) of LAMP amplification products on the sample pad of LFB and then depositing an aliquot of running buffer (150 μl) on the sample pad of LFB.

### Bacterial Strains and Clinical Sputum Samples

In this report, experimental bacteria, including reference strains H37Rv (ATCC 27294) and H37Ra (ATCC 25177), *M. bovis* (ATCC 19210), *M. bovis* Bacillus Calmette-Guerin (*M. bovis* BCG), *M. microti* (ATCC 19422), and 21 NTM strains were obtained from the Sichuan Provincial Center for Disease Control and Prevention, China. Ten isolates of MTB were isolated and provided by Guiyang Pulmonary Hospital (Guiyang, China). Nine strains of nonmycobacteria and two isolates of *M. bovis* were isolated by GuiZhou Provincial Center for Disease Control and Prevention, China (Table 1).

**TABLE 1:** The information of strains in this study.

**Bacteria^a^**	**Strain no. (source of strain)^b^**	**No. of strains**
MTB	H37Rv (ATCC 27294)	1
	H37Ra (ATCC 25177)	1
	Isolated strains (GZCDC)	10
*M. bovis*	ATCC 19210	1
	*Bacillus Calmette-Guerin* (BCG)	1
	Isolated strains (GZCDC)	2
*M. microti*	ATCC 19422	1
*M. intracellulare*	ATCC 13950	1
*M. aurum*	ATCC 23366	1
*M. smegmatis*	ATCC 19420	1
*M. parofortuitum*	ATCC 19686	1
*M. terrae*	ATCC 15755	1
*M. nonchromogenicum*	ATCC 19530	1
*M. aichiense*	ATCC 27280	1
*M. gilvum*	ATCC 43909	1
*M. marinum*	ATCC 927	1
*M. neoaurum*	ATCC 25795	1
*M. phlei*	ATCC 11758	1
*M. scrofulaceum*	ATCC 19981	1
*M. triviale*	ATCC 23290	1
*M. xenopi*	ATCC 19250	1
*M. ulcerans*	ATCC 19423	1
*M. malmoense*	ATCC 29571	1
*M. abscessus*	ATCC 19977	1
*M. fortuitum*	ATCC 6841	1
*M. gordonae*	ATCC 14470	1
*M. gastri*	ATCC 15754	1
*M. kansassi*	ATCC 12478	1
*Klebsiella pneumoniae*	Isolated strains (GZCDC)	1
*Pseudomonas aeruginosa*	Isolated strains (GZCDC)	1
*Listeria monocytogenes*	Isolated strains (GZCDC)	1
*Streptococcus pneumoniae*	Isolated strains (GZCDC)	1
*Staphylococcus aureus*	Isolated strains (GZCDC)	1
*Escherichia coli*	Isolated strains (GZCDC)	1
*Bacillus cereus*	Isolated strains (GZCDC)	1
*Enterococcus faecalis*	Isolated strains (GZCDC)	1
*Salmonella* spp.	Isolated strains (GZCDC)	1
Total		47

*^a^MTB, Mycobacterium tuberculosis.*

*^b^ATCC, American Type Culture Collection; GZCDC, Guizhou Provincial Center for Disease Control and Prevention.*

A total of 108 sputum samples (consisting of 84 samples of TB patients and 24 samples of non-TB patients) were collected from Guizhou Provincial Center for Disease Control and Prevention during the period of February 2019 to August 2019, and these samples were utilized to test the feasibility of our assay.

### Processing the Clinical Specimens

All sputum samples were divided into two groups (one group was utilized for the AFB smear and culture, and the other was utilized for the mLAMP-LFB and multiplex PCR assays by extracting their genomic DNA). These samples (group 1) were subjected to AFB smearing after staining with the Ziehl–Neelson stain according to routine steps and were cultured on modified L-J medium following the instructions (Zhu-hai Baso Biotechnology Co., Ltd., Guangdong, China) (Otu et al., 2008; Agrawal et al., 2016). All experiments were conducted in the Laboratory of Infectious Disease of Experimental Center, Guizhou Provincial Center for Disease Control and Prevention (Guiyang, China).

### DNA Extraction of Cultures and Clinical Specimens

Genomic DNA for 47 bacterial strains was extracted by the Bacterial Genomic DNA Extraction Kit (Takara Biomedical Technology Co., Ltd., Beijing, China), and their absorbances were tested at the A260/280 wavelengths using an ultramicro spectrophotometer (Thermo Fisher Scientific Co., Ltd., Beijing, China). The DNA templates were stored under –20°C. The templates of the MTB reference strain (H37Rv, ATCC 27294) were used for confirmation experiments, optimal temperature and time determinations, sensitivity analysis, and serial dilutions (12.5 ng, 1.25 ng, 125 pg, 12.5 pg, 1.25 pg, 125 fg, 12.5 fg, and 1.25 fg per microliter). One microliter aliquot of each gradient was used for the sensitivity analysis of the singlex and mLAMP assays.

All clinical samples, after handling with 4% NaOH and phosphate buffered saline (PBS, pH 6.8–7.2) and concentrating by centrifugation (4,000 rpm/min, 15 min), were centrifuged at 10,000 rpm/min for 10 min. Then, the pellets were washed twice with 70% ethanol, air-dried, suspended in 50 μl of TE (1×), and stored at −20°C. Isolation steps for genomic DNA were performed according to phenol/chloroform extraction and ethanol precipitation methods (van Soolingen et al., 1991). Three microliters of DNA templates were used for mLAMP-LFB and multiplex PCR assays (Kathirvel et al., 2014).

### Design and Synthesis of LAMP Primers

**FIGURE 1:**
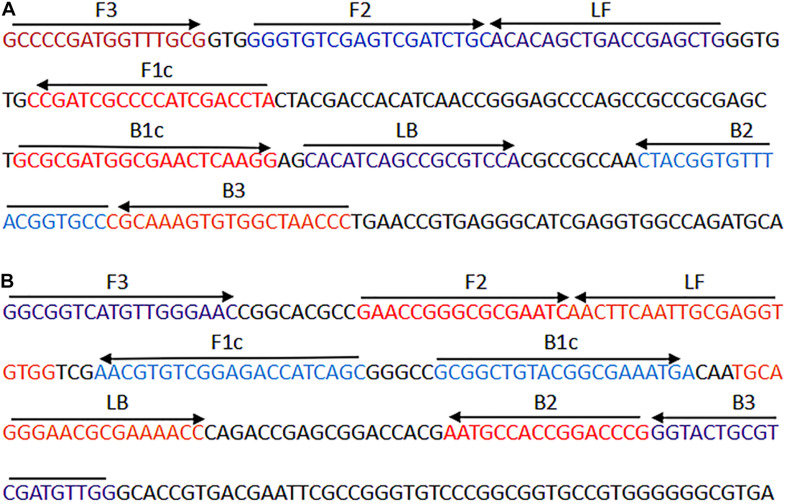
The sequence and location of the *IS6110* and *mtp40* genes were used to design mLAMP primers. **(A)** The nucleotide sequence of the sense strand of the *IS6110* gene is displayed. **(B)** The nucleotide sequence of the sense strand of the *mtp40* gene is shown. The sense and complementary sequences used are indicated by right arrows and left arrows. mLAMP, multiplex loop-mediated isothermal amplification.

The *IS6110* and *mtp40* genes were chosen as target sequences in our studies. After sequence alignment and screening using the Basic Local Alignment Search Tool (BLASTn), two sets of specific LAMP primers (FIP, BIP, LF, LB, F3, and B3) for the *IS6110* (GenBank accession no. X17348.1) and *mtp40* (GenBank accession no. S69737.1) genes were designed by LAMP primer design software (PrimerExplorer V5, Eiken Chemical Co., Ltd., Tokyo, Japan). 6-Carboxyfluorescein (6-FAM) was labeled on the 5′ end of the *IS6110-*FIP primer. Meanwhile, the 5′ end of the *mtp40*-FIP primer was labeled with digoxigenin (Dig). The primer information (e.g., sequence, length position, and modification of primer pairs) is shown in Figure 1 and Table 2. All primers (HPLC purification grade) were synthesized and purified by Tianyi-Huiyuan Biotech Co., Ltd. (Beijing, China).

**TABLE 2:** The primers used in the LAMP assay for the detection of *Mycobacterium tuberculosis.*

**Gene**	**Primer**	**Sequence (5′–3′)^a^**	**Length (nt)^b^**
*IS6110*	*IS*-F3	5′-GCCCCGATGGTTTGCG-3′	16
	*IS*-B3	5′-GGGTTAGCCACACTTTGCG-3′	19
	*IS*-FIP*	5′-FAM-TAGGTCGATGGGGCGATCGG-GGGTGTCGAGTCGATCTGC-3′	40
	*IS*-BIP	5′-GCGCGATGGCGAACTCAAGG-GGCACCGTAAACACCGTAG-3′	40
	*IS*-LF	CAGCTCGGTCAGCTGTGT-3′	18
	*IS*-LB	5′-CACATCAGCCGCGTCCA-3′	17
*mtp40*	*mtp*-F3	5′-GGCGGTCATGTTGGGAAC-3′	18
	*mtp*-B3	5′-CCAACATCGACGCAGTACC-3′	19
	*mtp*-FIP*	5′-Dig-GCTGATGGTCTCCGACACGTT-GAACCGGGCGCGAATC-3′	38
	*mtp*-BIP	5′-GCGGCTGTACGGCGAAATGA-CGGGTCCGGTGGCATT-3′	37
	*mtp*-LF	CCACACCTCGCAATTGAAGTT-3′	21
	*mtp*-LB	5′-TGCAGGGAACGCGAAAACC-3′	19

*^*a*^FAM, 6-carboxyfluorescein; Dig, digoxigenin.*

*^*b*^nt, nucleotide.*

### The Singlex LAMP Reaction

The availability of two sets of LAMP primers for the *IS6110* and *mtp40* genes was confirmed using the standard singlex LAMP assay with the follow-up test. The mixtures (25 μl) of singlex LAMP reaction included the following: 12.5 μl 2× isothermal amplification buffer (BF), 1 μl Bst 2.0 DNA polymerase (8 U), 1.6 μM each of FIP^∗^ and BIP, 0.8 μM each of LF and LB, 0.4 μM each of F3 and B3, 1 μl of 0.1 mM biotin-14-dCTP, 1 μl visual indicator (MG), templates (genome DNA for 1 μl of pure culture and 3 μl of samples), and double distilled water (ddH_2_O) were added to 25 μl. The mixtures were heated at 65°C for 1 h and then it was terminated at 85°C for 5 min. Then, the singlex LAMP amplification products were monitored and analyzed by using visual indicators, real-time turbidity, biosensor verification, and 1.5% agarose gel electrophoresis.

Then, the optimal reaction temperature of the two LAMP primers was determined by setting the amplification temperature range from 60 to 70°C (with an interval of 1°C), and the genomic DNA (12.5 pg/μl) of MTB (H37Rv, ATCC 27294) was used as templates in this experiment. The whole reaction was carried out at a constant temperature according to the standard singlex LAMP assay and monitored by employing a Loopamp real-time turbidimeter *LA-*500 (Eiken Chemical Co., Ltd.). Finally, the threshold value was 0.1, and a turbidity of >0.1 was judged as positive for the LAMP reaction by a real-time turbidimeter (Wang et al., 2019). The amplicons of the LAMP assay caused a color change from blue to light blue, while the negative controls and blank control became colorless by using visual detection (MG). The negative control (NC) containing 1 μl genomic DNA of the *Klebsiella pneumoniae* strain and the blank control (BC) containing 1 μl ddH_2_O were conducted in the singlex LAMP mixed system.

### The Standard mLAMP Assay

The mLAMP assay was performed in a 25-μl mixture containing 12.5 μl 2× isothermal amplification buffer (BF), 1 μl Bst 2.0 DNA polymerase (8 U), 1.4 μM each of *mtp*-FIP^∗^ and *mtp*-BIP, 0.7 μM each of *mtp*-LF and *mtp*-LB, 0.35 μM each of *mtp*-F3 and *mtp*-B3, 1.4 μM each of *IS*-FIP^∗^ and *IS*-BIP, 0.7 μM each of *IS*-LF and *IS*-LB, 0.35 μM each of *IS*-F3 and *IS*-B3, 1 μl of 0.1 mM biotin-14-dCTP, 1 μl visual indicator (MG), and template (genome DNA for 1 μl of pure culture or 3 μl of samples), and the ddH_2_O was added to 25 μl. The mixture was incubated at 65°C for 60 min and inactivated at 85°C for 5 min. The verification and analysis methods for the mLAMP amplification products were performed using biosensors and visual reagents.

Moreover, the reaction time of the mLAMP assay was optimized by setting the range from 10 to 80 min (10 min intervals) according to the optimal temperature of singlex LAMP tests. These results were analyzed by biosensors and visual detection reagents. Template DNA (12.5 pg/μl) of MTB (H37Rv, ATCC 27294) was employed, and reaction tubes without template DNA were used as blank controls (ddH_2_O) in our studies.

### Verification of Sensitivity and Specificity of the LAMP-LFB Assays

The analytical sensitivity of the singlex LAMP and mLAMP methods was confirmed by using serial dilutions (12.5 ng to 1.25 fg per microliter) of the purified genomic DNA of MTB (H37Rv, ATCC 27294) and conducting the assays using a defined number of replicates (usually 20 per dilution). The limit of detection (LoD) of the experiment was defined as the lowest concentration of genomic DNA that, when detected by serial dilutions, resulted in the detection of MTB in ≥95% of the tests performed in this study (Chakravorty et al., 2017). According to the standard LAMP assay, the sensitivity tests were verified by biosensors, visual indicators, 1.5% agarose gel electrophoresis, and real-time turbidity, and all assays were independently conducted in multiple replicates.

To analyze the specificity of the mLAMP-LFB assay, according to the abovementioned optimal reaction conditions, we carried out genomic DNA tests for 47 bacterial strains (Table 1), and 1 μl of template DNA was added to the reaction mixture. All mLAMP-LFB results were reported by the biosensor.

### Applicability of the mLAMP-LFB Assay to Sputum Samples

The feasibility of the mLAMP-LFB assay was validated with the following procedures. The culture of the MTB H37Rv reference strain (ATCC 25177) was homogenized by using a multichannel rapid mixer (Zhu-hai Yinke Medical Engineering Co., Ltd., Guangdong, China) for 1 min in sterile distilled water (10 mg/ml). The bacterial suspension was used to prepare a series of 10-fold dilutions in sterile distilled water (10^–1^ to 10^–8^), and the dilutions were inoculated on a plate including Middlebrook 7H10 agar (Difco, Franklin Lakes, NJ, United States) and OADC reagents (oleic acid–albumin–dextrose–catalase; Becton, Dickinson and Company, Sparks, MD, United States). The numbers of CFUs were counted after incubation at 37°C for 4 weeks according to a previous study (Dauendorffer et al., 1999). Then, the aliquots (100 μl) of each suspension with suitable dilutions (10^–1^ to 10^–8^) were simultaneously added to sputum samples (900 μl), which were collected from healthy persons, and the numbers of MTB were adjusted to approximately 4.8 × 10^6^, 4.8 × 10^5^, 4.8 × 10^4^, 4.8 × 10^3^, 4.8 × 10^2^, 4.8 × 10^1^, 4.8 × 10^0^, and 4.8 × 10^–1^ CFU/ml. After adding serial dilutions of quantitative CFU to the specimens, the detection sensitivity of mLAMP-LFB assays was confirmed by extracting genomic DNA from these artificial sputum samples following the steps for processing clinical specimens. A total of 3 μl DNA templates were used for the mLAMP-LFB assays. Sputum samples without MTB were selected as negative controls. The analysis was performed three times for each test.

In addition, all sputum samples were subjected to conventional methods (i.e., AFB smear, modified L-J media), the multiplex PCR and mLAMP-LFB methods, and their genomic DNA was extracted according to a previous description (van Soolingen et al., 1991). The multiplex PCR assay, referencing a previous publication (Kathirvel et al., 2014), was performed in a 50-μl mixture containing 25 μl 2 × Taq Master Mix (CoWin Biosciences Co., Ltd., Beijing, China); 0.2 μM *IS*-F (5′-GTGAG GGCATCGAGGTGG-3′), *IS*-R (5′-CGTAGGCGTCGGTCA CAAA-3′), *mtp*-F (5′-GGTTCCCAACACCACGTTAG-3′), and *mtp*-R (5′-CCAACATCGACGCAGTACC-3′); 3 μl of specimen DNA; and double distilled water, which was added to 50 μl. The amplifications were conducted in an automated thermal cycler (Thermo Fisher Scientific Co., Ltd., Beijing, China). The samples were denatured at 94°C for 2 min, and 30 amplification cycles were performed. The cycles consisted of denaturation at 94°C (30 s), annealing at 60°C (30 s), and primer extension at 72°C (30 s). The final extension time was set for 2 min. The PCR products were visualized in a 1.5% agarose gel with ethidium bromide staining under UV light (Bio-Rad, Hercules, CA, United States). The mLAMP-LFB test was implemented as described above. The detection results for multiplex PCR and mLAMP-LFB assays are shown in Table 3.

**TABLE 3:** Determination of the detection results for the multiplex PCR and mLAMP-LFB assays.

**Detection results**	**Multiplex PCR/mLAMP-LFB^a^**		**Species types^b^**
	***IS6110***	***mtp40***	
Positive	+	+	P1
	−	+	P1
	+	−	P2
Negative	−	−	

*^*a*^PCR, polymerase chain reaction; mLAMP, multiplex loop-mediated isothermal amplification; LFB, lateral flow biosensor; +, positive amplification; −, negative amplification.*

*^*b*^P1, *Mycobacterium tuberculosis*; P2, other members of *Mycobacterium tuberculosis* complex (excluding *Mycobacterium tuberculosis*).*

### Statistical Analysis

SPSS software (ver. 26.0; IBM, Armonk, NY, United States) was used to calculate and analyze the sensitivity, specificity, positive predictive value (PPV), and negative predictive value (NPV) of the tests in this study.

## Results

### Confirmation and Detection of LAMP-LFB Products

**FIGURE 2:**
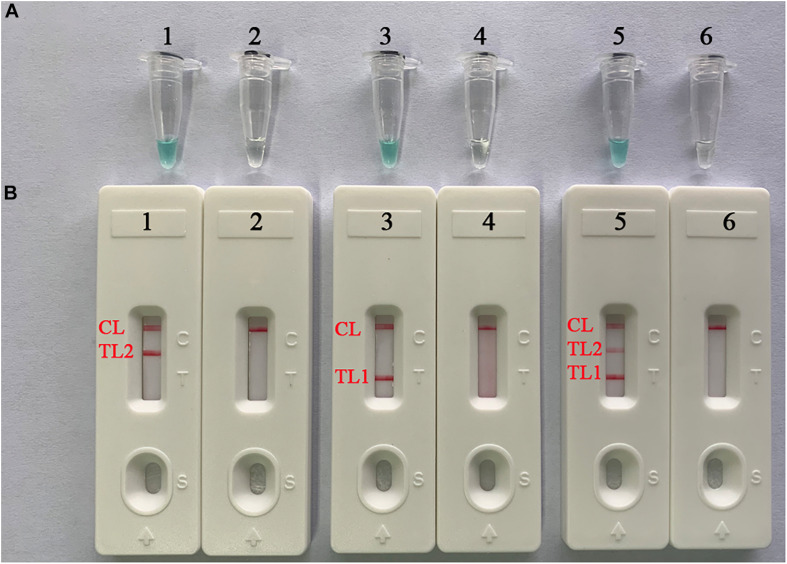
Confirmation and detection of singlex LAMP- and mLAMP-LFB products. **(A)** Color change of singlex LAMP and mLAMP tubes. **(B)** The biosensor applied for rapid verification of singlex LAMP and mLAMP amplicons. Tube 1 (biosensor 1): positive amplification of *mtp40*-LAMP for the reference strain MTB (H37Rv, ATCC 27294); tube 2 (biosensor 2): negative control (*Klebsiella pneumoniae*); tube 3 (biosensor 3): positive amplification of *IS6110*-LAMP for the reference strain MTB; tube 4 (biosensor 4): negative control (*Klebsiella pneumoniae*); tube 5 (biosensor 5): positive amplification of mLAMP for the reference strain MTB; tube 6 (biosensor 6): negative control (*Klebsiella pneumoniae*). mLAMP, multiplex loop-mediated isothermal amplification; TL1, positive amplification of the *IS6110* sequence; TL2, positive amplification of the *mtp40* gene; CL, control line.

To verify the effectiveness of the two sets of primers, we implemented confirmation tests for the singlex LAMP and mLAMP assays using genomic DNA extracted from pure cultures of MTB. The color of the positive amplification changed from blue to light blue, while the negative control was colorless (Figure 2A). Strips were employed to verify the reaction results by observing the color change of lines (i.e., test lines and/or control line) with the naked eye. For positive amplification, the red bands of the control line (CL) and test line 1 (TL1 for *IS6110* detection) or test line 2 (TL2 for *mtp40* detection) were seen in single LAMP; CL, TL1, and TL2 were red bands in mLAMP experiments, but only CL was shown red in the negative control (Figure 2B).

### Optimization of Application Temperature and Time for LAMP-LFB Assay

**FIGURE 3:**
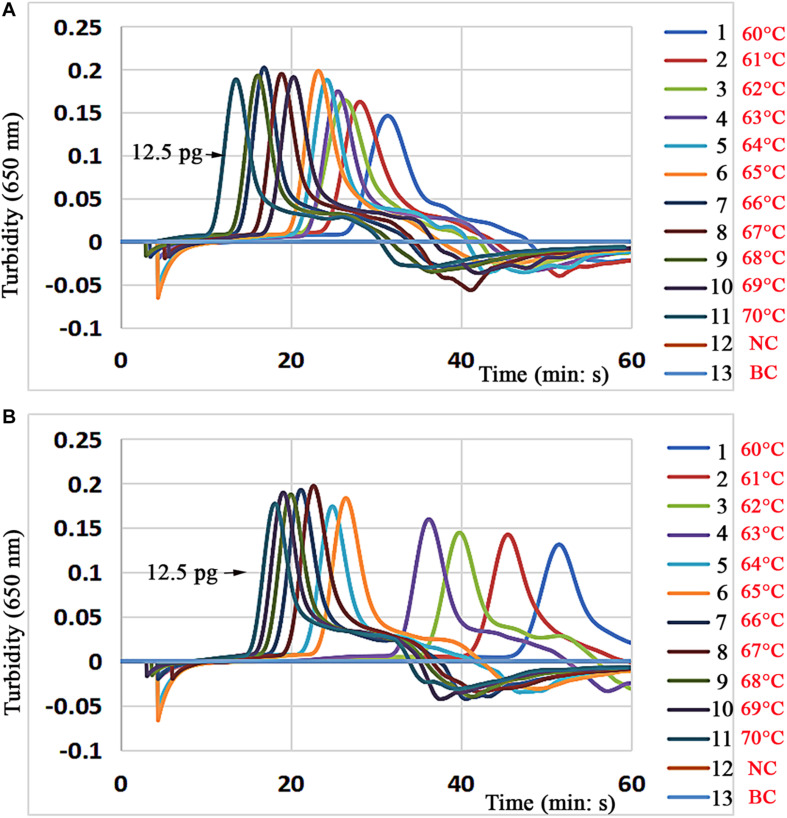
Optimal reaction temperature for the LMAP assay. The standard singlex LAMP reactions of the *IS6110* **(A)** and *mtp40* **(B)** genes for the detection of MTB (H37Rv, ATCC 27294) were monitored by real-time turbidimetry, and the corresponding curves of DNA concentrations are marked in the figures. This threshold value was 0.1, and a turbidity >0.1 was considered positive amplification. A total of 11 kinetic graphs (1–11) were generated at various temperatures (60–70°C, 1°C intervals) with target pathogen DNA at the level of 12.5 pg per reaction. **(A)** The graphs from 4 to 10 displayed higher amplification efficiency; **(B)** the graphs from 5 to 11 showed higher amplification efficiency; **(A,B)** the lines (12–13) were negative control and blank control. LAMP, loop-mediated isothermal amplification.

To achieve better amplification efficiency, the reaction temperature of the LAMP-LFB assays was optimized according to standard conditions, and different incubation temperatures were performed for the singlex LAMP tests ranging from 60 to 70°C with an interval of 1°C. The MTB reference strain (H37Rv, ATCC 27294) was used at the level of 12.5 pg per mixture, the reactions were monitored and analyzed by real-time turbidity, and the optimal reactions occurred with temperatures of 66°C for the singlex LAMP experiments (Figures 3A,B).

**FIGURE 4:**
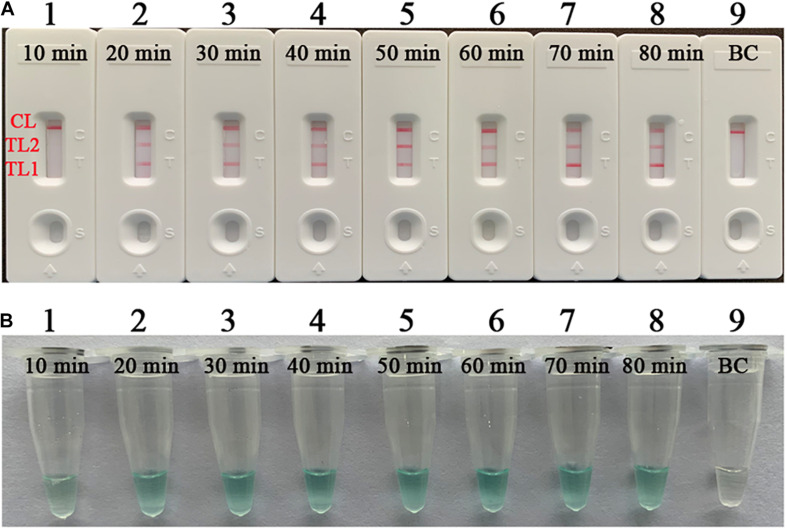
Optimal reaction time for the mLAMP-LFB assay. The standard mLAMP-LFB test for the detection of MTB (H37Rv, ATCC 27294) was verified by visual reagent (MG) and LFB. **(A)** Biosensor applied for the detection of mLAMP amplicons. **(B)** Color change of the LAMP tubes. These drawings of biosensors (1–8) and tubes (1–8) were confirmed at diverse times (10 to 80 min, 10 min intervals) with template DNA at the level of 12.5 pg per microliter; and biosensor 9 and tube 9: blank control (ddH_2_O). Reactions 2–8 displayed effective and stable amplification. LFB, lateral flow biosensor; MG, malachite green; mLAMP, multiplex loop-mediated isothermal amplification; ddH_2_O, double distilled water; TL1, positive amplification of the *IS6110* sequence; TL2, positive amplification of the *mtp40* gene; CL, control line.

In addition, the experiments determining the optimal reaction time for mLAMP-LFB showed that the amplification efficiency from 20 to 80 min was relatively stable, and the reaction time was made as short as possible while still obtaining detection (Figures 4A,B). Thus, an amplification temperature of 66°C and a time of 40 min were implemented for the rest of the singlex LMAP-LFB and mLAMP-LFB assays in the current report.

### Sensitivity of Singlex LAMP-LFB Assay

**FIGURE 5:**
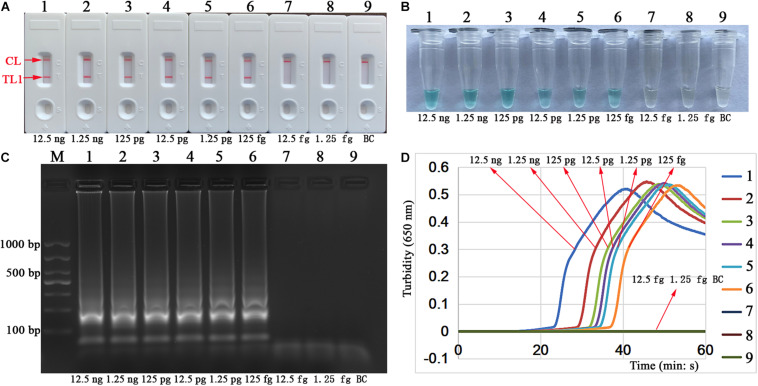
Sensitivity of a singlex LAMP assay for the *IS6110* gene. The tests were conducted according to the standard singlex LAMP reaction, and serial dilutions (12.5 ng, 1.25 ng, 125 pg, 12.5 pg, 1.25 pg, 125 fg, 12.5 fg, and 1.25 fg per microliter) of the target template DNA were implemented in the LAMP reaction. A total of four verification methods, namely, biosensor **(A)**, MG indicator **(B)**, 1.5% agarose gel electrophoresis **(C)**, and real-time turbidity **(D)**, were applied to analyze the *IS6110*-LAMP amplicons. Biosensors **(A)**/tubes **(B)**/lanes **(C)**/turbidity **(D)** 1–8 correspond to genomic DNA of MTB (H37Rv, ATCC 27294) from 12.5 ng/μl to 1.25 fg/μl; biosensor/tube/lane/turbidity 9: blank control (ddH_2_O). Line M: DL 1000 DNA maker. MG, malachite green; LAMP, loop-mediated isothermal amplification; ddH_2_O, double distilled water; TL1, positive amplification of the *IS6110* sequence; TL2, positive amplification of the *mtp40* gene; CL, control line.

**FIGURE 6:**
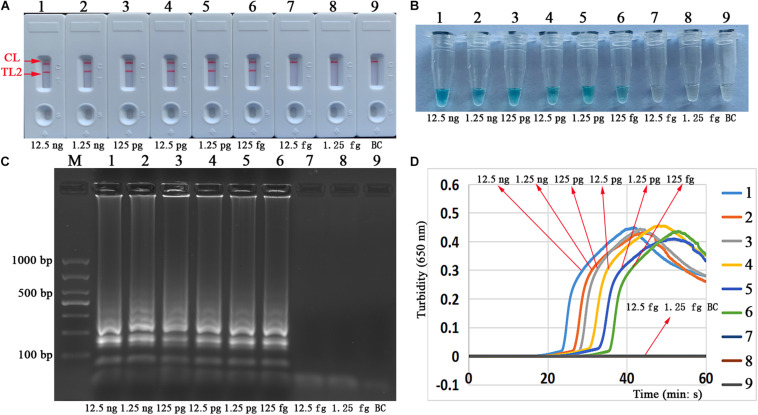
Sensitivity of a singlex LAMP assay for the *mtp40* gene. The tests were conducted according to the standard singlex LAMP reaction, and serial dilutions (12.5 ng, 1.25 ng, 125 pg, 12.5 pg, 1.25 pg, 125 fg, 12.5 fg, and 1.25 fg per microliter) of the target template DNA were implemented in the LAMP reaction. A total of four verification methods, namely, biosensor **(A)**, MG indicator **(B)**, 1.5% agarose gel electrophoresis **(C)**, and real-time turbidity **(D)**, were applied to analyze the *mtp40*-LAMP amplicons. Biosensors **(A)**/tubes **(B)**/lanes **(C)**/turbidity **(D)** 1–8 correspond to genomic DNA of MTB (H37Rv, ATCC 27294) from 12.5 ng/μl to 1.25 fg/μl; biosensor/tube/lane/turbidity 9: blank control (ddH_2_O). Line M: DL 1000 DNA maker. MG, malachite green; LAMP, loop-mediated isothermal amplification; ddH_2_O, double distilled water; TL1, positive amplification of the *IS6110* sequence; TL2, positive amplification of the *mtp40* gene; CL, control line.

We performed and analyzed the detection sensitivity of singlex LAMP-LFB by testing serial dilutions of the genomic DNA of purified culture MTB in this study. The assay LoDs of *IS6110*-LAMP and *mtp40*-LAMP experiments were 125 fg of template DNA per reaction. The confirmed LFB results (Figures 5A, 6A) were consistent with the visual reagent (Figures 5B, 6B), 1.5% agarose gel electrophoresis results (Figures 5C, 6C), and real-time turbidimeter (Figures 5D, 6D).

### Sensitivity of the mLAMP-LFB Assay

**FIGURE 7:**
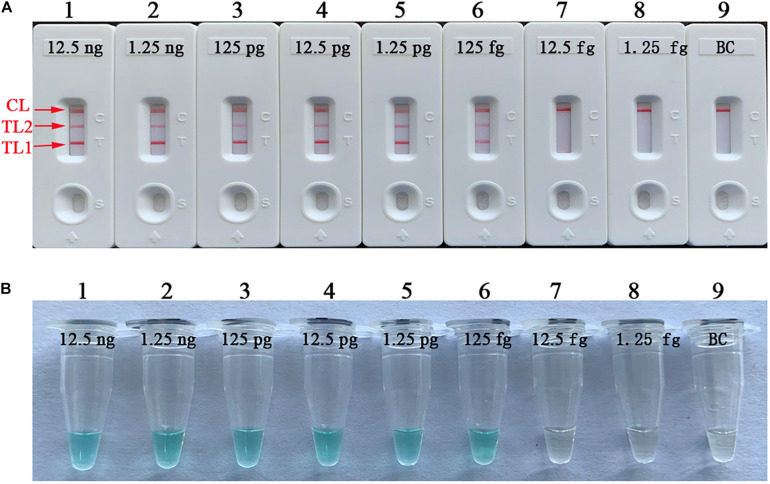
Analytical sensitivity of the mLAMP assay targeting the *IS6110* and *mtp40* genes. Two sets of specific LAMP primers were added simultaneously to a mixture, and the sensitivity of the mLAMP assay for the detection of MTB (H37Rv, ATCC 27294) was analyzed by using the biosensor **(A)** and colorimetric indicator **(B)**. Biosensors (1–8) and tubes (1–8) represent DNA levels of 12.5 ng, 1.25 ng, 125 pg, 12.5 pg, 1.25 pg, 125 fg, 12.5 fg, and 1.25 fg per microliter; biosensor (9) and tube (9): blank control (ddH_2_O). mLAMP, multiplex loop-mediated isothermal amplification; ddH_2_O, double distilled water; TL1, positive amplification of the *IS6110* sequence; TL2, positive amplification of the *mtp40* gene; CL, control line.

The analytical sensitivity of the mLAMP assay can also reach 125 fg per reaction tube. The simultaneous appearance of three crimson bands (CL, TL1, and TL2) on the biosensor indicated positive amplification of the *IS6110* and *mtp40* genes, while only the CL band was red in the absence of amplification and the negative control (Figure 7A). The visual indicator can distinguish the positive and negative reactions through the color change of reaction tubes (Figure 7B).

### Analytical Specificity of the mLAMP-LFB Assay

**FIGURE 8:**
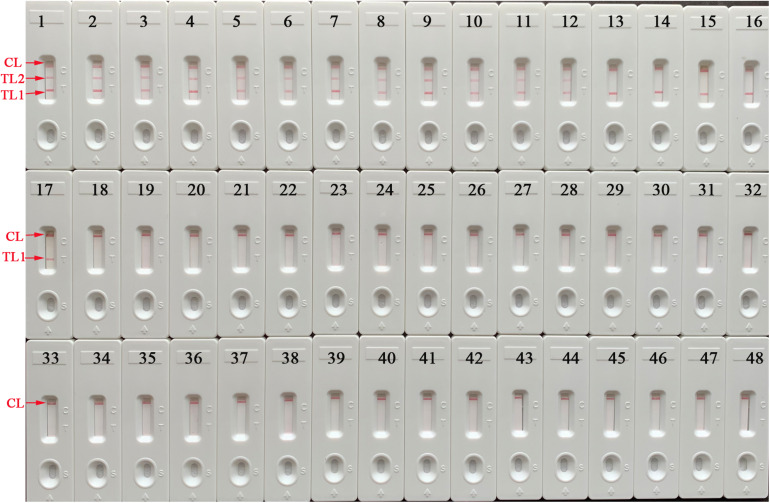
Specificity of the mLAMP assay for various pathogens. The mLAMP tests were implemented using different template DNA samples and monitored by using a lateral flow biosensor. Biosensors 1–2, strains of MTB H37Rv (ATCC 27294) and H37Ra (ATCC 25177); biosensors 3–12, MTB isolates; biosensors 13–14, strains of *M. bovis* (ATCC 19210) and *M. bovis* BCG; biosensors 15–16, isolates of *M. bovis*; biosensors 17, *M. microti*; biosensors 18–38, strains of *M. intracellulare*, *M. aurum*, *M. smegmatis*, *M. parofortuitum*, *M. terrae*, *M. nonchromogenicum*, *M. aichiense*, *M. gilvum*, *M. marinum*, *M. neoaurum*, *M. phlei*, *M. scrofulaceum*, *M. triviale*, *M. xenopi*, *M. ulcerans*, *M. malmoense*, *M. abscessus*, *M. fortuitum*, *M. gordonae*, *M. gastri*, *M. kansassi*; biosensors 39–46, strains of *Pseudomonas aeruginosa*, *Listeria monocytogenes*, *Streptococcus pneumoniae*, *Staphylococcus aureus*, *Escherichia coli*, *Bacillus cereus*, *Enterococcus faecalis*, *Salmonella* spp.; biosensors 47–48, negative control (*Klebsiella pneumoniae*) and blank control (ddH_2_O). mLAMP, multiplex loop-mediated isothermal amplification; ddH_2_O, double distilled water; BCG, Bacillus Calmette–Guerin; TL1, positive amplification of the *IS6110* sequence; TL2, positive amplification of the *mtp40* gene; CL, control line.

The specificity of mLAMP-LFB assays was evaluated with the optimal amplification temperature and time by extracting genomic DNA from 26 mycobacterial reference strains, 10 isolates of MTB, 2 isolates of *M. bovis*, and 9 nonmycobacterial strains (Table 1). The analytical specificity of the mLAMP-LFB examined was 100% in our studies. Test line 1, test line 2, and the control line appeared simultaneously as red bands on the biosensor, indicating positive reactions for MTB strains had occurred (including H37Rv, H37Ra, and isolates), whereas only TL1 and CL were shown on the strips, indicating positive results for *M. bovis* (ATCC 19210), *M. bovis* BCG, isolates of *M. bovis*, and *M. microti* (ATCC 19422). Only a red band (CL) appeared, indicating negative results for NTMs, nonmycobacterial strains, the negative control, and the blank control (Figure 8). Thus, the mLAMP-LFB method established here was able to detect typical strains of MTBC (i.e., reference strains of MTB and *M. bovis*, isolates of MTB and *M. bovis*, *M. bovis* BCG, and *M. microti*) and simultaneously differentiate MTB from other MTBC members in our studies.

### Practical Application of the mLAMP-LFB Assay for Sputum Samples

**FIGURE 9:**
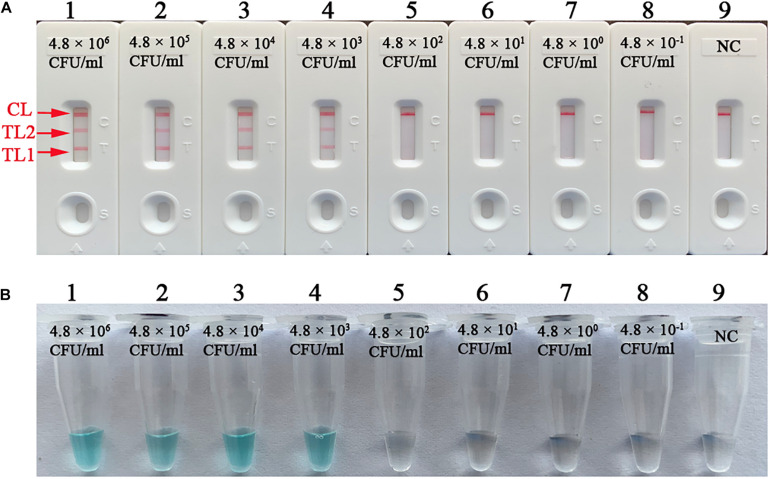
Practical sensitivity of the mLAMP assay for the detection of sputum samples. The practical sensitivity of the mLAMP assay for the detection of artificial specimens was analyzed by using the biosensor **(A)** and colorimetric indicator **(B)**. Biosensors (1–8) and tubes (1–8) represent 4.8 × 10^6^, 4.8 × 10^5^, 4.8 × 10^4^, 4.8 × 10^3^, 4.8 × 10^2^, 4.8 × 10^1^, 4.8 × 10^0^, and 4.8 × 10^– 1^ CFU/ml; biosensor (9) and tube (9): negative control. The detection limit of mLAMP-LFB for the examination of sputum samples was 4.8 × 10^3^ CFU/ml (∼14.4 CFU/reaction). mLAMP, multiplex loop-mediated isothermal amplification; TL1, positive amplification of the *IS6110* sequence; TL2, positive amplification of the *mtp40* gene; CL, control line.

The practical sensitivity of the mLAMP assay was 4.8 × 10^3^ CFU/ml (∼14.4 CFU per reaction). The simultaneous appearance of three crimson bands (CL, TL1, and TL2) on the biosensor indicated positive amplification of the *IS6110* and *mtp40* genes, while only the CL band displayed red in the absence of amplification and the negative control (Figure 9A). The visual indicator can distinguish the positive and negative reactions through the color change of reaction tubes (Figure 9B).

In addition, for 84 sputum samples of TB cases, 45 specimens were AFB smear-positive and 39 were negative; 73 samples were culture-positive and 11 were negative as determined by standard culture methods (modified L-J medium); 79 samples were multiplex PCR-positive and 5 were negative, of which 76 were amplification-positive for *IS6110* and *mtp40* genes and 3 amplification-positive for only the *mtp40* gene; and by mLAMP-LFB assay, 82 sputum samples were examination-positive, which included 79 incubation-positive for *IS6110* and *mtp40* genes (CL, TL1, and TL2) and 3 incubation-positive for only the *mtp40* gene (CL and TL2), and two samples were negative (CL) (Supplementary Table 1). For 24 sputum samples of non-TB cases, the results for the four detection methods were negative (Supplementary Table 2). The sensitivity of mLAMP-LFB (97.62%) was higher than those of the AFB smear (47.56%), modified L-J media (86.90%), and multiplex PCR (94.05%) tests in the detection of clinical samples (Table 4). Thus, these data indicated that our mLAMP-LFB assay employed in this study had better detection capabilities for clinical specimens than conventional methods and/or multiplex PCR.

**TABLE 4:** Sensitivity and specificity of four methods for examination results of sputum samples.

**Methods^a^**	**Sputum samples^b^**		**Sensitivity (%)**	**Specificity (%)**	**PPV^c^ (%)**	**NPV^d^ (%)**
	**TB cases (*N* = 84)**	**Non-TB cases (*N* = 24)**				
AFB smear						
Positive	39	0	47.56	100	100	34.78
Negative	45	24				
L-J culture						
Positive	73	0	86.90	100	100	68.57
Negative	11	24				
Multiplex PCR						
Positive	79	0	94.05	100	100	82.76
Negative	5	24				
mLAMP-LFB						
Positive	82	0	97.62	100	100	92.31
Negative	2	24				

*^*a*^AFB, acid-fast bacilli; L-J, Lowenstein–Jensen medium; PCR, polymerase chain reaction; mLAMP, multiplex loop-mediated isothermal amplification; LFB, lateral flow biosensor.*

*^*b*^TB, tuberculosis.*

*^*c*^PPV, positive predictive value.*

*^*d*^NPV, negative predictive value.*

## Discussion

Tuberculosis is still the infectious disease with the highest morbidity and mortality in the world, and its main pathogen is MTB, but other MTBC members (particularly *M. bovis*) should not be ignored (Ansumana et al., 2017). Thus, rapid, accurate, and sensitive differentiation of MTB from other MTBC members is important for preventing and controlling the spread of TB epidemics. However, traditional detection methods (i.e., AFB smear, L-J culture, and biochemical tests) usually cannot meet the requirements necessary for rapid detection in terms of time and sensitivity. PCR and PCR-based assays (e.g., real-time quantitative PCR and GeneXpert techniques) and immunologic tests (e.g., T-SOPT TB and enzyme-linked immunosorbent assays) can achieve rapid detection for MTB, but the special instrument requirements and/or expensive reagents hinder their application in the field and in resource-poor areas (Gallo et al., 2016; Lekhak et al., 2016; Rao et al., 2021). Among a wide variety of rapid examination methods, LAMP, as a low-cost and valuable technique, has been widely used in the detection of various pathogens, including viruses, bacteria, and fungi (Han et al., 2007; Hsu et al., 2017). Nevertheless, the verification and analysis of LAMP products (including visual reagents, agarose gel electrophoresis, and real-time turbidimetry) have always been pivotal issues. Unfortunately, with these analytical methods, it is difficult to distinguish between specific and nonspecific amplifications and verify the products of simultaneous amplification for two target genes in a single reaction (Wang et al., 2019).

The biosensor, as a recognition technique for two labeled nucleic acid amplicons, was designed and developed to be widely utilized in the analysis of LAMP products (Wang et al., 2017, 2018). The result can be verified by visually observing the color change of TL and/or CL after aliquots (1.2 μl) of LAMP amplicons and aliquots (150 μl) of running buffer are added to the sample pad. The analysis time of LAMP products was greatly shortened using LFB (1–2 min). Compared with other measurement methods (colorimetric indicators, agarose gel electrophoresis, and real-time turbidity), the LFB method was not only fast and sensitive but also easy to operate and less error-prone, especially as it reduces the requirements of special instruments, reagents, and additional verification steps. In our experiments, the previous double-labeling method was replaced by biotin-14-dCTP (single-labeled FIP primer applied), which greatly reduced the possibility of cross-reaction between the two sets of primers (Table 2).

Previously, many studies have confirmed that using the *IS6110* sequence (several copies exist in the MTBC genome) as a target for the LAMP reaction obtained the preferable sensitivity and specificity for the detection of typical MTBC strains in the diagnosis of TB (Singh et al., 2004; Joon et al., 2017; Sreedeep et al., 2020). Recently, a diagnostic method has achieved rapid detection of MTBC based on mLAMP combined with single-labeled LFB but could not distinguish MTB from other members of MTBC due to the limitations of gene selection and LFB design (Wang et al., 2021). Thus, there is a necessary requirement to recognize another target gene (*mtp40*) that can distinguish MTB, and it was used as an accurate molecular marker in our study (Jia et al., 2012; Kathirvel et al., 2014). Therefore, to achieve a more efficient diagnosis, a mLAMP-LFB assay targeting the *IS6110* and *mtp40* genes was designed and performed successfully to distinguish MTB from other members of MTBC in this report.

To determine the optimal reaction conditions, different reaction temperatures and times were employed while conducting the mLAMP-LFB test. The LAMP-LFB assays showed higher amplification efficiency at 66°C than at other temperatures and displayed stable results when the amplification time ranged from 20 to 80 min when using LFB. In the specificity tests (mLAMP-LFB was applied), the positive results were reference MTB (H37Rv and H37Ra), *M. bovis*, *M. bovis* BCG, *M. microti*, and isolates of MTB and *M. bovis*, while NTMs and nonmycobacterial species were completely excluded. In particular, there were three crimson lines (TL1, TL2, and CL) for the MTB strains (H37Rv, H37Ra, and isolates), which confirmed that the mLAMP-LFB assay performed had a high degree of specificity for the differentiation of MTB from other members (*M. bovis*, *M. bovis* BCG, *M. microti*, and isolates of *M. bovis*) in our studies (Table 2 and Figure 8). In addition to its high specificity, the newly developed mLAMP-LFB was able to detect 125 fg genomic DNA per reaction tube for pure culture of MTB and 4.8 × 10^3^ CFU/ml artificial sputum samples (∼14.4 CFU per reaction) (Figures 7, 9). The technique had a higher sensitivity than that of the conventional PCR method (10–100-fold), which only detects 1–10 pg/μl (Jia et al., 2012).

In this report, although the four confirmatory methods (biosensor, visual reagents, real-time turbidimeter, and 1.5% agarose gel electrophoresis) were identical in detecting LAMP amplicons, the LFB was more operable in terms of convenience, accuracy, and instrument requirements. Since this mLAMP-LFB only needs to be incubated in an isothermal environment of 66°C for 40 min, many thermostatic instruments can be used for amplification procedures (e.g., dry block heater, portable battery-powered device, and thermostat water bath), which is specifically suitable for point-of-care and field laboratories.

In this report, to evaluate the practical applicability of the mLAMP-LFB assay, clinical specimens (TB and non-TB patients) were tested by using comparative approaches (containing conventional methods and multiplex PCR). The mLAMP-LFB experiment showed excellent sensitivity (97.62%) and specificity (100%), especially for 82 strains of MTB (containing 73 strains cultured positive with L-J medium that were identified as MTB by p-nitrobenzoic acid medium and thiophene 2-carboxylic acid hydrazide medium; data not shown) that were screened accurately, which provided the possibility to accurately diagnose TB and guide clinical medication (Supplementary Table 1). When compared with AFB smears and conventional culture, the rate of positive samples detected by mLAMP-LFB was higher. The reasons for this may be due to (i) the limitation of sample quality (e.g., impurities and bacterial number) and/or the proficiency of the testing, (ii) the presence of dead and/or nonculturable bacilli in samples, and (iii) false positives in mLAMP-LFB assays; however, the probability of false positives in mLAMP-LFB is low because the experiments involved the specific labeled primer and LFB verification (Agrawal et al., 2016; Wang et al., 2018). Interestingly, two samples (collected from TB cases) tested negative by four detection methods, which may be caused by incorrect collection of specimens and/or the lack of *IS6110* (including three examination-positive specimens) and *mtp40* elements in the bacilli genome (Kathirvel et al., 2014; Gallo et al., 2016). Although culture methods are often regarded as the “standard” for MTB detection/identification, they are time-consuming (4–8 weeks) and inefficient and easily delay the optimal opportunity of the patient for treatment. Thus, these data indicated that the established mLAMP-LFB has a high degree of sensitivity and specificity for the detection of clinical specimens and is a potentially valuable diagnostic tool for TB.

Furthermore, the whole procedure of implementing the mLAMP-LFB test, including specimen processing (35 min), isothermal reaction (40 min), and verification (1–2 min), could be completed within 80 min. The cost of a single mLAMP-LFB reaction was estimated to be approximately 5 USD, and the value of LFB was 2.5 USD per test. Malachite Green was used during the mLAMP-LFB experiment, and the preliminary judgment was made directly by the naked eye after the amplification process was completed, which eliminated the complicated detection steps.

## Conclusion

In this report, mLAMP-LFB targeting the *IS6110* and *mtp40* genes was successfully developed and implemented. In the process of application and evaluation, the mLAMP-LFB method exhibited excellent specificity and sensitivity by examining reference strains and clinical specimens. The biosensor, which could intuitively reflect the detection results and eliminate the use of a complicated apparatus, was applied to verify the mLAMP amplicons in our studies. Hence, the mLAMP-LFB assay developed is a convenient, rapid, sensitive, and reliable technique that was able to detect representative strains of MTBC and differentiate MTB simultaneously from other MTBC members, and it can be regarded as a potential screening tool for TB in clinical, basic, field, and resource-poor areas.

## Data Availability Statement

The original contributions presented in the study are included in the article/Supplementary Material, further inquiries can be directed to the corresponding author/s.

## Author Contributions

XY and SLi conceived and designed this study. SLi supervised the study. XY, SLi, SLe, JH, XC, ZX, XW, YC, WZ, WC, HC, and YH conducted the experiments. XY, SLi, JH, and ZX analyze the data. SLi, SLe, JH, XC, YC, WZ, WC, HC, and YH contributed the reagents, analysis tools, and contributed the materials. XY performed the software and drafted the manuscript. SLi revised the manuscript. All authors contributed to the article and approved the submitted version.

## Conflict of Interest

The authors declare that the research was conducted in the absence of any commercial or financial relationships that could be construed as a potential conflict of interest.

## Publisher’s Note

All claims expressed in this article are solely those of the authors and do not necessarily represent those of their affiliated organizations, or those of the publisher, the editors and the reviewers. Any product that may be evaluated in this article, or claim that may be made by its manufacturer, is not guaranteed or endorsed by the publisher.

## Abbreviations

TB: tuberculosis
MTBC: *Mycobacterium tuberculosis*
MTBC: *Mycobacterium tuberculosis* complex
LAMP: loop-mediated isothermal amplification
WHO: World Health Organization
LFB: lateral flow biosensor
mLAMP-LFB: multiplex loop-mediated isothermal amplification linked to a nanoparticle-based lateral flow biosensor
PCR: polymerase chain reaction
ATCC: American Type Culture Collection
LoD: limit of detection
MG: malachite green
GZCDC: Guizhou Provincial Center for Disease Control and Prevention
FAM: carboxy fluorescein
Dig: digoxigenin
nt: nucleotide
CL: control line
TL1: test line 1
TL2: test line 2
DW: distilled water.
