# Supplementary material for: Rapid and Visual Differentiation of Mycobacterium tuberculosis From the Mycobacterium tuberculosis Complex Using Multiplex Loop-Mediated Isothermal Amplification Coupled With a Nanoparticle-Based Lateral Flow Biosensor
Source: Front Microbiol. 2021 Aug 2;12:708658. doi: 10.3389/fmicb.2021.708658 (PMC8365424; doi:10.3389/fmicb.2021.708658)
Supplement: Supplementary file 1 [file Data_Sheet_1.docx]

Supplementary Material

# Supplementary Tables

- **Supplementary Table 1** Detection results of AFB smear, multiplex PCR, mLAMP-LFB and culture for 84 sputum samples.

| **Patient**  **type ^a^** | **Sample**  **number** | **AFB smear ^b^** | **multiplex PCR** | | **multiplex PCR ^c^** | **mLAMP-LFB** | | **mLAMP-LFB ^d^** | **Culture ^e^** |
| --- | --- | --- | --- | --- | --- | --- | --- | --- | --- |
|  |  |  | **IS6110** | **mtp40** |  | **IS6110** | **mtp40** |  |  |
| TB | LPS01 | P | + | + | P | + | + | P | P |
| TB | LPS02 | N | - | - | N | + | + | P | N |
| TB | LPS03 | N | + | + | P | + | + | P | P |
| TB | LPS04 | N | + | + | P | + | + | P | P |
| TB | LPS05 | N | + | + | P | + | + | P | P |
| TB | LPS06 | P | + | + | P | + | + | P | P |
| TB | LPS07 | N | + | + | P | + | + | P | P |
| TB | LPS08 | P | + | + | P | + | + | P | P |
| TB | LPS09 | N | + | + | P | + | + | P | P |
| TB | LPS10 | P | + | + | P | + | + | P | P |
| TB | LPS11 | P | + | + | P | + | + | P | P |
| TB | LPS12 | P | + | + | P | + | + | P | P |
| TB | LPS13 | P | + | + | P | + | + | P | P |
| TB | LPS14 | P | + | + | P | + | + | P | P |
| TB | LPS15 | P | + | + | P | + | + | P | P |
| TB | LPS16 | N | + | + | P | + | + | P | P |
| TB | LPS17 | N | - | - | N | - | - | N | N |
| TB | LPS18 | P | + | + | P | + | + | P | P |
| TB | LPS19 | N | + | + | P | + | + | P | P |
| TB | LPS20 | N | + | + | P | + | + | P | P |
| TB | LPS21 | P | - | + | P | - | + | P | P |
| TB | LPS22 | P | + | + | P | + | + | P | P |
| TB | LPS23 | P | + | + | P | + | + | P | P |
| TB | LPS24 | N | + | + | P | + | + | P | P |
| TB | LPS25 | P | + | + | P | + | + | P | P |
| TB | LPS26 | P | + | + | P | + | + | P | P |
| TB | LPS27 | N | - | - | N | + | + | P | N |
| TB | LPS28 | P | + | + | P | + | + | P | P |
| TB | LPS29 | N | + | + | P | + | + | P | P |
| TB | LPS30 | P | + | + | P | + | + | P | P |
| TB | LPS31 | N | + | + | P | + | + | P | P |
| TB | LPS32 | N | + | + | P | + | + | P | N |
| TB | LPS33 | P | + | + | P | + | + | P | P |
| TB | LPS34 | P | + | + | P | + | + | P | P |
| TB | LPS35 | P | + | + | P | + | + | P | P |
| TB | LPS36 | N | + | + | P | + | + | P | P |
| TB | LPS37 | N | - | - | N | + | + | P | N |
| TB | LPS38 | N | + | + | P | + | + | P | P |
| TB | LPS39 | N | + | + | P | + | + | P | P |
| TB | LPS40 | N | + | + | P | + | + | P | P |
| TB | LPS41 | P | + | + | P | + | + | P | P |
| TB | LPS42 | N | + | + | P | + | + | P | N |
| TB | LPS43 | P | + | + | P | + | + | P | P |
| TB | LPS44 | P | + | + | P | + | + | P | P |
| TB | LPS45 | P | - | + | P | - | + | P | P |
| TB | LPS46 | P | + | + | P | + | + | P | P |
| TB | LPS47 | P | + | + | P | + | + | P | P |
| TB | LPS48 | P | + | + | P | + | + | P | N |
| TB | LPS49 | P | + | + | P | + | + | P | P |
| TB | LPS50 | N | + | + | P | + | + | P | P |
| TB | LPS51 | P | + | + | P | + | + | P | P |
| TB | LPS52 | N | + | + | P | + | + | P | P |
| TB | LPS53 | N | + | + | P | + | + | P | P |
| TB | LPS54 | N | + | + | P | + | + | P | N |
| TB | LPS55 | N | + | + | P | + | + | P | P |
| TB | LPS56 | N | + | + | P | + | + | P | P |
| TB | LPS57 | N | + | + | P | + | + | P | P |
| TB | LPS58 | N | + | + | P | + | + | P | P |
| TB | LPS59 | N | + | + | P | + | + | P | P |
| TB | LPS60 | P | + | + | P | + | + | P | P |
| TB | LPS61 | N | + | + | P | + | + | P | P |
| TB | LPS62 | N | + | + | P | + | + | P | P |
| TB | LPS63 | N | - | + | P | - | + | P | P |
| TB | LPS64 | N | + | + | P | + | + | P | P |
| TB | GY01 | P | + | + | P | + | + | P | P |
| TB | GY02 | P | + | + | P | + | + | P | P |
| TB | GY03 | P | + | + | P | + | + | P | P |
| TB | GY04 | P | + | + | P | + | + | P | P |
| TB | GY05 | P | + | + | P | + | + | P | P |
| TB | GY06 | N | + | + | P | + | + | P | P |
| TB | GY07 | N | + | + | P | + | + | P | N |
| TB | GY08 | N | + | + | P | + | + | P | P |
| TB | GY09 | N | + | + | P | + | + | P | N |
| TB | GY10 | N | + | + | P | + | + | P | P |
| TB | GY11 | N | + | + | P | + | + | P | P |
| TB | GY12 | N | - | - | N | - | - | N | N |
| TB | GY13 | P | + | + | P | + | + | P | P |
| TB | GY14 | P | + | + | P | + | + | P | P |
| TB | GY15 | P | + | + | P | + | + | P | P |
| TB | GY16 | N | + | + | P | + | + | P | P |
| TB | GY17 | N | + | + | P | + | + | P | P |
| TB | GY18 | P | + | + | P | + | + | P | P |
| TB | GY19 | N | + | + | P | + | + | P | P |
| TB | GY20 | N | + | + | P | + | + | P | P |

P, positive; N, negative. +, positive amplification; -, negative amplification.

^a^ TB, Tuberculosis.

^b^ AFB, acid-fast bacilli.

^c^ PCR, polymerase chain reaction.

^d^ mLAMP, multiplex loop-mediated isothermal amplification; LFB, lateral flow biosensor.

^e^ Culture, modified Lowenstein-Jensen.

- **Supplementary Table 2** Detection results of AFB smear, multiplex PCR, mLAMP-LFB and culture for 24 sputum samples.

| **Patient**  **Type ^a^** | **Sample**  **number** | **AFB smear ^b^** | **multiplex PCR** | | **multiplex PCR ^c^** | **mLAMP-LFB** | | **mLAMP-LFB ^d^** | **Culture ^e^** |
| --- | --- | --- | --- | --- | --- | --- | --- | --- | --- |
|  |  |  | **IS6110** | **mtp40** |  | **IS6110** | **mtp40** |  |  |
| Non-TB | AS01 | N | - | - | N | - | - | N | N |
| Non-TB | AS02 | N | - | - | N | - | - | N | N |
| Non-TB | AS03 | N | - | - | N | - | - | N | N |
| Non-TB | AS04 | N | - | - | N | - | - | N | N |
| Non-TB | AS05 | N | - | - | N | - | - | N | N |
| Non-TB | AS06 | N | - | - | N | - | - | N | N |
| Non-TB | AS07 | N | - | - | N | - | - | N | N |
| Non-TB | AS08 | N | - | - | N | - | - | N | N |
| Non-TB | AS09 | N | - | - | N | - | - | N | N |
| Non-TB | AS10 | N | - | - | N | - | - | N | N |
| Non-TB | AS11 | N | - | - | N | - | - | N | N |
| Non-TB | AS12 | N | - | - | N | - | - | N | N |
| Non-TB | AS13 | N | - | - | N | - | - | N | N |
| Non-TB | AS14 | N | - | - | N | - | - | N | N |
| Non-TB | AS15 | N | - | - | N | - | - | N | N |
| Non-TB | AS16 | N | - | - | N | - | - | N | N |
| Non-TB | AS17 | N | - | - | N | - | - | N | N |
| Non-TB | AS18 | N | - | - | N | - | - | N | N |
| Non-TB | AS19 | N | - | - | N | - | - | N | N |
| Non-TB | AS20 | N | - | - | N | - | - | N | N |
| Non-TB | AS21 | N | - | - | N | - | - | N | N |
| Non-TB | AS22 | N | - | - | N | - | - | N | N |
| Non-TB | AS23 | N | - | - | N | - | - | N | N |
| Non-TB | AS24 | N | - | - | N | - | - | N | N |

P, positive; N, negative. +, positive amplification; -, negative amplification.

^a^ TB, Tuberculosis.

^b^ AFB, acid-fast bacilli.

^c^ PCR, polymerase chain reaction.

^d^ mLAMP, multiplex loop-mediated isothermal amplification; LFB, lateral flow biosensor.

^e^ Culture, modified Lowenstein-Jensen.
